# Supplementary figures and images for: Naturally-associated bacteria modulate Orsay virus infection of Caenorhabditis elegans
Source: PLoS Pathog. 2024 Jan 17;20(1):e1011947. doi: 10.1371/journal.ppat.1011947 (PMC10824439; doi:10.1371/journal.ppat.1011947)

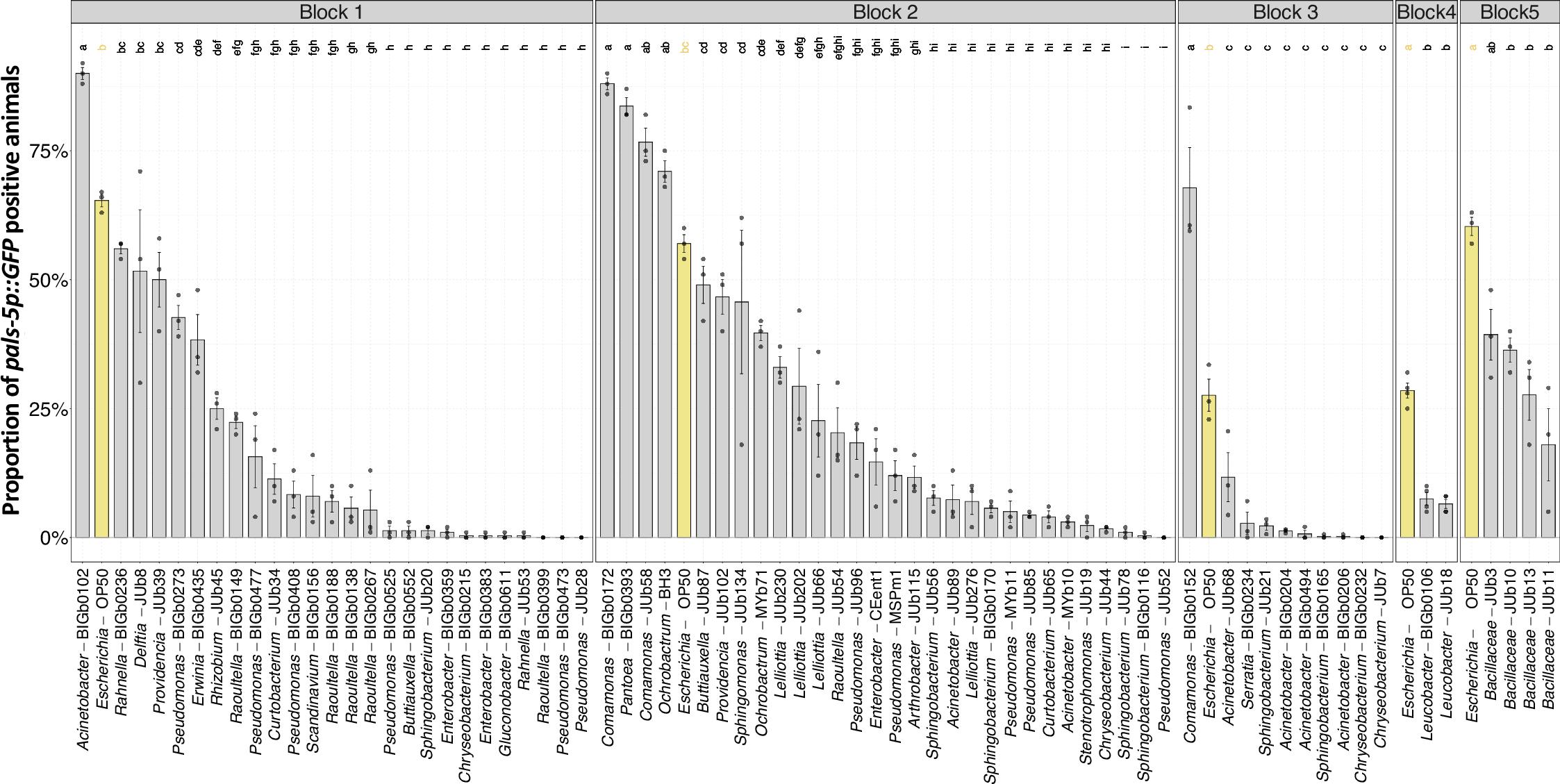

Supplement: S1 Fig — Original data used in Fig 1, including here the experimental block structure. For each bacterial environment, three replicates of ca. 100 ERT54 animals were challenged with the OrV JUv1580. The proportion of animals activating the pals-5p::GFP reporter was measured 72 hpi. Data are presented as mean ± standard error. Letters over the bars indicate letter-based grouping for multiple comparisons. The yellow bar indicates E. coli OP50. (TIF) [file ppat.1011947.s001.tif]

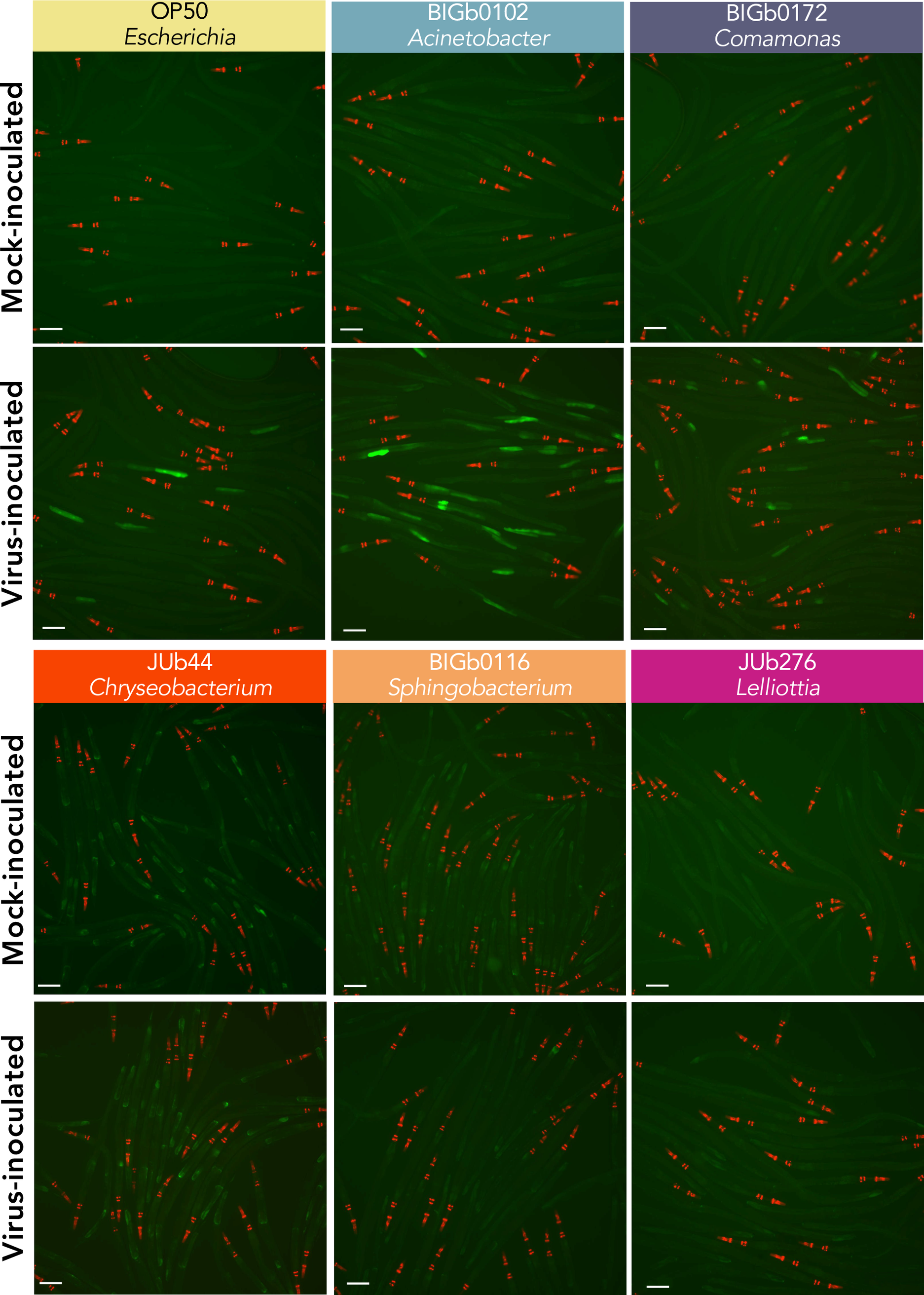

Supplement: S2 Fig — Animals mock-inoculated with M9 or inoculated with OrV JUv1580 were visualized at 72 hpi using a 10x objective mounted on AxioImager M1 (Zeiss) compound microscope and images were captured with a PIXIS 1024 (Princeton instruments) camera. Scale bar represents 100 μm. GFP-positive animals can be seen in the intestinal cells of virus-inoculated animals on the three bacteria on top. Note that a less intense fluorescence can be seen in the posterior intestinal cells of animals in both mock- and virus-inoculated populations, especially in JUb44 environments. This posterior intestinal fluorescence is also observed in uninfected animals grown in OP50 [39]. We hypothesize that this fluorescence is caused by the 3’UTR of unc-54 used in plasmids that generated the ERT54 strain. This DNA fragment probably contains cis-regulatory sites for the adjacent gene, aex-5, expressed in posterior intestinal cells as mentioned in [67]. (TIF) [file ppat.1011947.s002.tif]

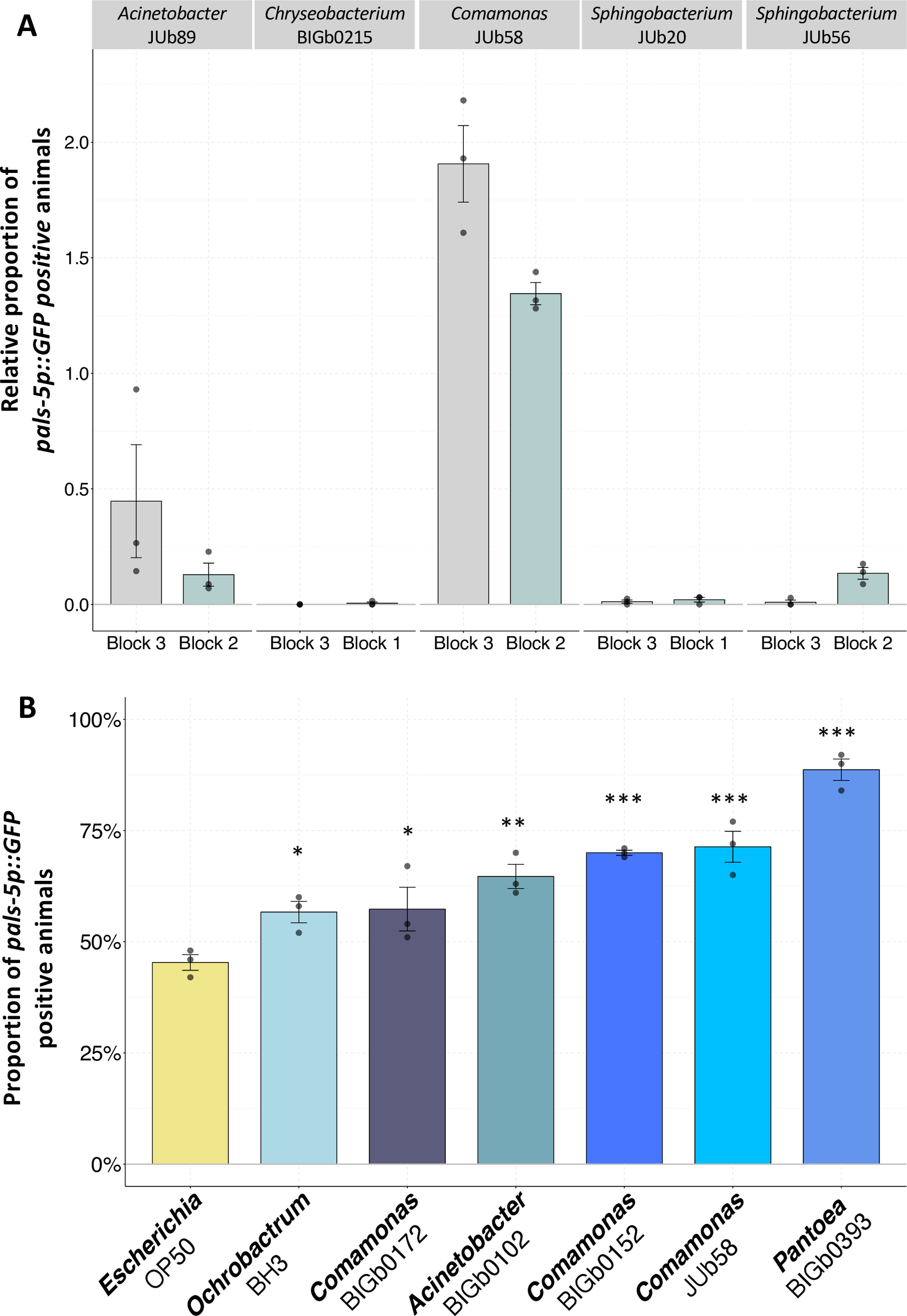

Supplement: S3 Fig — (A) The impact of bacterial strains on viral infection of ERT54 nematodes is consistent across experimental blocks (performed on different days). (B) New experiment with the bacteria that enhanced infection on the initial screening shown in Fig 1. Each data point represents a biological replicate, with 100 animals assayed per population. Data are presented as mean ± standard error. Asterisks on the graphs represent values of significance: *** P < 0.001; ** P < 0.01; * 0.01 < P < 0.05; P values higher than 0.05 are not labeled. Significance was calculated using a general linear model with bacteria as a factor and Dunnett’s contrasts to compare all conditions against the Escherichia OP50 reference. (TIF) [file ppat.1011947.s003.tif]

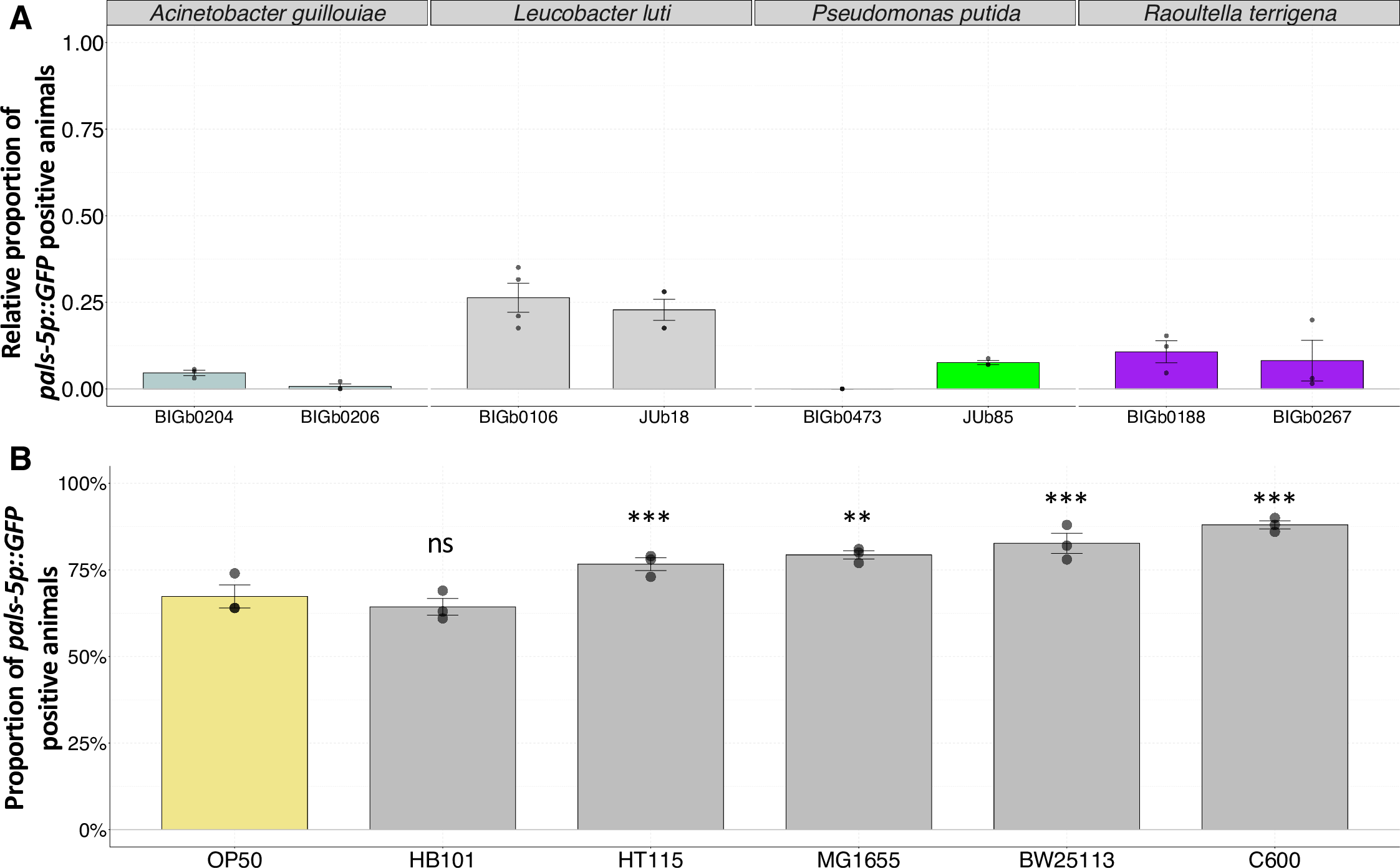

Supplement: S4 Fig — (A) Activation of the pals-5p::GFP reporter upon viral infection of ERT54 animals on different strains of the same bacterial species. (B) Activation of pals-5p::GFP reporter upon viral infection of animals on different E. coli strains. Each data point represents a biological replicate, with 100 animals assayed per population. Data are presented as mean ± standard error. Asterisks on the graphs represent values of significance: *** P < 0.001; ** P < 0.01; * 0.01 < P < 0.05; P values higher than 0.05 are not labeled. Significance was calculated using a general linear model with bacteria as a factor and Dunnett’s contrasts to compare all conditions against the Escherichia OP50 reference. (TIF) [file ppat.1011947.s004.tif]

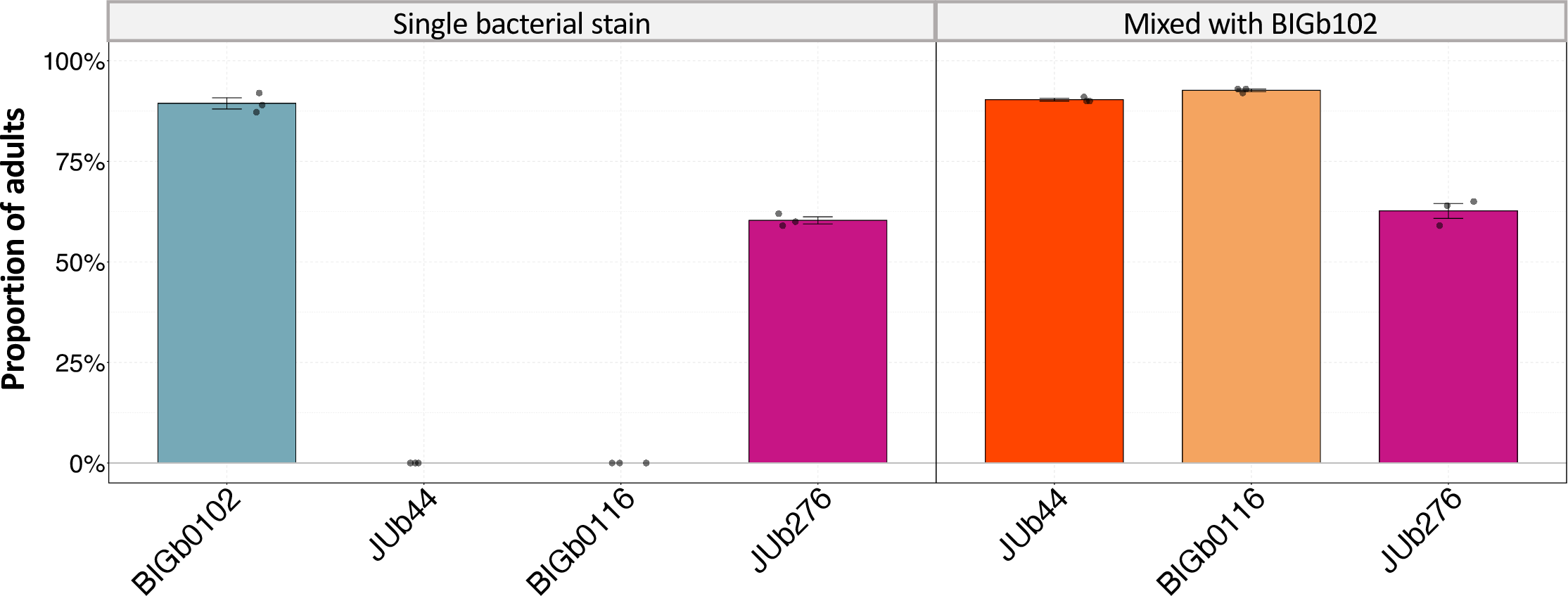

Supplement: S5 Fig — Arrested axenic L1 larvae of the ERT54 strain were exposed to each monobacterial environment or to mixed environments composed of 20% of BIGb0102 and 80% of another bacterium (indicated in the x axis). The proportion of adults of 3 independent populations (100 animals per population) per environmental condition was observed after 46 and 62 h. (TIF) [file ppat.1011947.s005.tif]

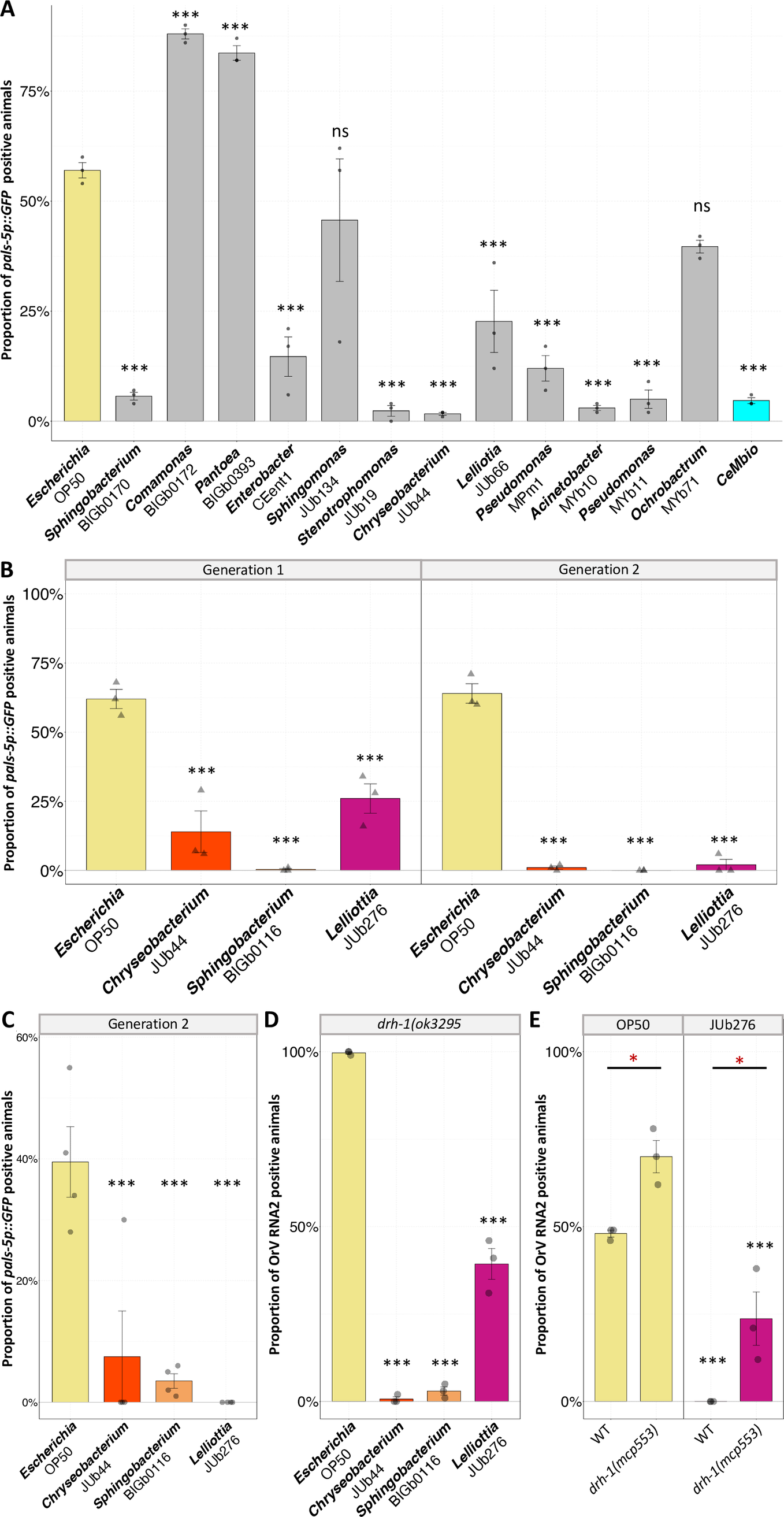

Supplement: S6 Fig — (A) Activation of the pals-5p::GFP reporter upon OrV infection of animals on different single CeMbio strains and the whole CeMbio community. (B) Repetition of experiment shown in Fig 6, but only evaluating the activation of the pals-5p::GFP reporter. (C) Repetition of experiment shown in Fig 6, using a transfer of an agar chunk after 2 generations (detailed in methods). (D) Repetition of experiments, testing drh-1 animals, shown in Fig 8D. (E) Susceptibility to viral infection on Lelliottia JUb276 of animals carrying a natural deletion allele of drh-1. Data are presented as mean ± standard error. Black symbols indicate the significance of the difference between the labeled bacteria and the Escherichia OP50 reference, while red symbols indicate the significance of differences between genotypes: *** P < 0.001; ** P < 0.01; * 0.01 < P < 0.05; P values higher than 0.05 are labeled as “ns”. Significance was calculated using an analysis of variance with bacteria as a factor (Panels A,C,D), a general linear model where the factors were bacteria and generations (Panel D), or a general linear model where the factors were bacteria and host genotype (Panel E). In both cases Tukey contrasts were used for post hoc analyses. (TIF) [file ppat.1011947.s006.tif]

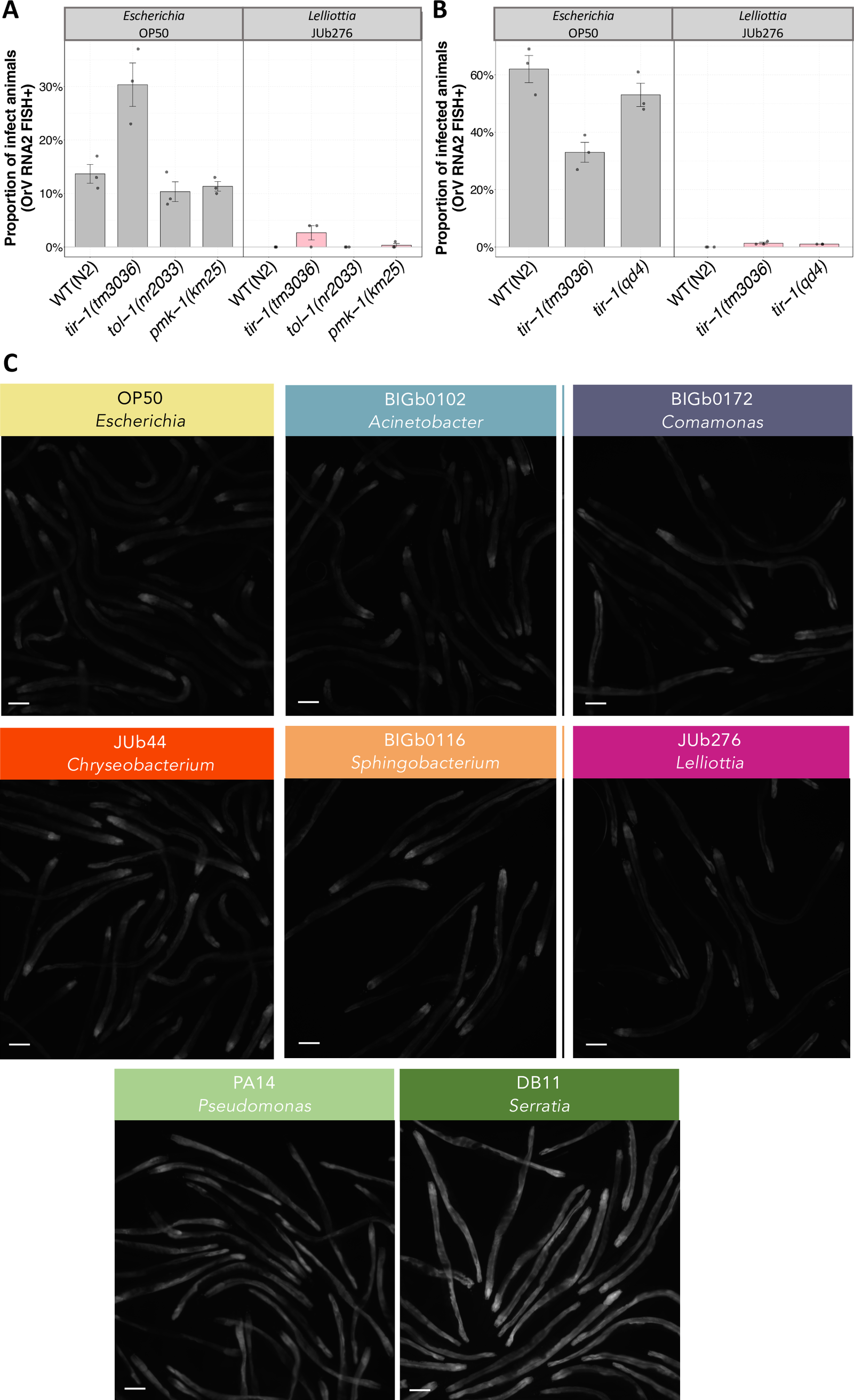

Supplement: S7 Fig — (A) Experiment testing, on Escherichia OP50 or Lelliottia JUb276, virus susceptibility of mutants with alterations in different genes involved in the response against bacterial infections. We tested three biological replicates per genotype, with 100 animals assayed per population. Data are presented as mean ± standard error. (B) Experiment testing, on Escherichia OP50 or Lelliottia JUb276, virus susceptibility of tir-1 mutants. We tested three biological replicates per genotype, with 100 animals assayed per population. Data are presented as mean ± standard error. (C) Fluorescence microscopy of the sysm-1p::GFP reporter. JU4289 animals carry the agsl219[sysm-1p::GFP + ttx-3p::GFP] transgene. Three day-old JU4289 animals grown at 20°C in the indicated bacterial environment were observed using a 10x objective on an AxioImager M1 (Zeiss) compound microscope. The Cherry and GFP fluorescence channels were captured with a PIXIS 1024 (Princeton instruments) camera and overlaid. PA14 and DB11 bacterial environments serve as positive control for the reporter activation. Scale bar represents 100 μm. (TIF) [file ppat.1011947.s007.tif]
