# Supplementary material for: Naturally-associated bacteria modulate Orsay virus infection of Caenorhabditis elegans
Source: PLoS Pathog. 2024 Jan 17;20(1):e1011947. doi: 10.1371/journal.ppat.1011947 (PMC10824439; doi:10.1371/journal.ppat.1011947)
Supplement: S1 Table — The pals-5p::GFP column indicates that C. elegans is able to activate the pals-5p::GFP reporter on the tested strains after heat shock. For this test, 48 hours-old ERT54 animals were placed at 30°C for 24 h, being observed right after the 24 h heat shock. FR is an abbreviation for France, GER for Germany, and USA for United States of America. (DOCX) [file ppat.1011947.s008.docx]

**Supplementary Table 1.** List of bacterial strains used in this work. The *pals-5p::GFP* column indicates that *C. elegans* is able to activate the *pals-5p::GFP* reporter on the tested strains after heat shock. For this test, 48 hours-old ERT54 animals were placed at 30 ºC for 24 h, being observed right after the 24 h heat shock. FR is an abbreviation for France, GER for Germany, and USA for United States of America.

| **Strain** | **Sampling location** | **16S best blast** | ***pals-5p::GFP* after heat shock** | **Reference** |
| --- | --- | --- | --- | --- |
| OP50 | - | *Escherichia coli* | Activated | 53 |
| BH3 | Franconville, FR | *Ochrobactrum sp.* | Activated | 38 |
| BIGb0102 | Orsay, FR | *Acinetobacter sp.* | Activated | 36 |
| BIGb0106 | Orsay, FR | *Leucobacter luti* | Activated | 16 |
| BIGb0116 | Orsay, FR | *Sphingobacterium sp.* | Activated | 16 |
| BIGb0138 | Orsay, FR | *Raoultella sp.* | Activated | 16 |
| BIGb0149 | Orsay, FR | *Raoultella sp.* | Not tested | 16 |
| BIGb0152 | Orsay, FR | *Comamonas sp.* | Activated | 16 |
| BIGb0156 | Orsay, FR | *Scandinavium goeteborgense* | Not tested | 16 |
| BIGb0165 | Orsay, FR | *Sphingobacterium sp.* | Activated | 16 |
| BIGb0170 | Orsay, FR | *Sphingobacterium multivorum* | Activated | 16 |
| BIGb0172 | Orsay, FR | *Comamonas piscis* | Activated | 36 |
| BIGb0188 | Orsay, FR | *Raoultella terrigena* | Not tested | 36 |
| BIGb0204 | Orsay, FR | *Acinetobacter sp.* | Not tested | 16 |
| BIGb0206 | Orsay, FR | *Acinetobacter guillouiae* | Activated | 16 |
| BIGb0215 | Orsay, FR | *Chryseobacterium nakagawai* | Not tested | 16 |
| BIGb0232 | Orsay, FR | *Chryseobacterium sp.* | Not tested | 16 |
| BIGb0234 | Orsay, FR | *Serratia sp.* | Activated | 16 |
| BIGb0236 | Orsay, FR | *Rahnella sp.* | Activated | 16 |
| BIGb0267 | Santeuil, FR | *Raoultella terrigena* | Activated | 16 |
| BIGb0273 | Santeuil, FR | *Pseudomonas brenneri* | Activated | 36 |
| BIGb0359 | Ivry, FR | *Enterobacter sp.* | Activated | 16 |
| BIGb0383 | Ivry, FR | *Enterobacter sp.* | Activated | 16 |
| BIGb0393 | Ivry, FR | *Pantoea nemavictus* | Activated | 16 |
| BIGb0399 | Ivry, FR | *Raoultella sp.* | Activated | 16 |
| BIGb0408 | Ivry, FR | *Pseudomonas sp.* | Activated | 16 |
| BIGb0435 | Ivry, FR | *Erwinia rhapontici* | Activated | 16 |
| BIGb0473 | Ivry, FR | *Pseudomonas putida* | Activated | 16 |
| BIGb0477 | Ivry, FR | *Pseudomonas psychrophila* | Not tested | 16 |
| BIGb0494 | Ivry, FR | *Acinetobacter johnsonii* | Activated | 16 |
| BIGb0525 | Ivry, FR | *Pseudomonas helmanticensis* | Activated | 16 |
| BIGb0552 | Ivry, FR | *Buttiauxella sp.* | Activated | 16 |
| BIGb0611 | Santeuil, FR | *Gluconobacter cerinus* | Activated | 36 |
| CEent1 | California, USA | *Enterobacter hormaechei* | Activated | 14 |
| JUb10 | Le Perreux, FR | *Bacillus sp.* | Not tested | 16 |
| JUb102 | Santeuil, FR | *Providencia alcalifaciens* | Activated | 16 |
| JUb11 | Le Perreux, FR | *Bacillus sp.* | Not tested | 16 |
| JUb115 | Santeuil, FR | *Arthrobacter sp.* | Activated | 16 |
| JUb13 | Le Perreux, FR | *Bacillus sp.* | Not tested | 16 |
| JUb134 | Santeuil, FR | *Sphingomonas molluscorum* | Activated | 41 |
| JUb18 | South Africa | *Leucobacter luti* | Activated | 16 |
| JUb19 | Le Blanc, FR | *Stenotrophomonas indicatrix* | Activated | 16 |
| JUb20 | Primel, FR | *Sphingobacterium sp.* | Activated | 36 |
| JUb202 | Le Blanc, FR | *Lelliottia sp.* | Not tested | 41 |
| JUb21 | Primel, FR | *Sphingobacterium sp.* | Activated | 16 |
| JUb230 | Plurien, FR | *Lelliottia sp.* | Activated | 41 |
| JUb276 | New Zealand | *Lelliottia sp.* | Activated | 37 |
| JUb28 | Santeuil, FR | *Pseudomonas protegens* | Activated | 16 |
| JUb3 | Le Perreux, FR | *Bacillus sp.* | Not tested | 16 |
| JUb34 | Santeuil, FR | *Curtobacterium sp.* | Activated | 16 |
| JUb39 | Santeuil, FR | *Providencia sp.* | Activated | 16 |
| JUb44 | Santeuil, FR | *Chryseobacterium scophthalmum* | Activated | 16 |
| JUb45 | Santeuil, FR | *Neorhizobium sp.* | Activated | 16 |
| JUb52 | Santeuil, FR | *Pseudomonas sp.* | Activated | 16 |
| JUb53 | Santeuil, FR | *Rahnella sp.* | Activated | 16 |
| JUb54 | Santeuil, FR | *Raoultella ornithinolytica* | Activated | 16 |
| JUb56 | Santeuil, FR | *Sphingobacterium sp.* | Activated | 16 |
| JUb58 | Santeuil, FR | *Comamonas sp.* | Activated | 16 |
| JUb65 | Santeuil, FR | *Curtobacterium flaccumfaciens* | Activated | 36 |
| JUb66 | Santeuil, FR | *Lelliottia amnigena* | Not tested | 16 |
| JUb68 | Santeuil, FR | *Acinetobacter sp.* | Not tested | 16 |
| JUb7 | Santeuil, FR | *Chryseobacterium sp.* | Activated | 16 |
| JUb78 | Santeuil, FR | *Sphingobacterium sp.* | Activated | 16 |
| JUb8 | Santeuil, FR | *Delftia acidovorans* | Activated | 16 |
| JUb85 | Santeuil, FR | *Pseudomonas putida* | Activated | 16 |
| JUb87 | Santeuil, FR | *Buttiauxella sp.* | Activated | 16 |
| JUb89 | Santeuil, FR | *Acinetobacter calcoaceticus* | Activated | 36 |
| JUb96 | Santeuil, FR | *Pseudomonas sp.* | Activated | 16 |
| MSPm1 | California, USA | *Pseudomonas berkeleyensis* | Activated | 41 |
| MYb10 | Kiel, GER | *Acinetobacter guillouiae* | Activated | 15 |
| MYb11 | Kiel, GER | *Pseudomonas lurida* | Activated | 15 |
| MYb71 | Kiel, GER | *Ochrobactrum vermis* | Activated | 15 |
| PA14 | - | *Pseudomonas aeruginosa* | Not tested |  |
| DB11 | - | *Serratia marcescens* | Not tested |  |
| CeMBio | Microbial community | | Activated | 41 |
