# Supplementary material for: Naturally-associated bacteria modulate Orsay virus infection of Caenorhabditis elegans
Source: PLoS Pathog. 2024 Jan 17;20(1):e1011947. doi: 10.1371/journal.ppat.1011947 (PMC10824439; doi:10.1371/journal.ppat.1011947)
Supplement: S2 Table — (DOCX) [file ppat.1011947.s009.docx]

S

**Supplementary Table 2.** Nematode strains used in this study.

| **Strain** | **Genotype** |
| --- | --- |
| ERT54 | *jyIs8[pals-5p::GFP; myo-2p::mCherry]* |
| ERT71 | *jyIs15[F26F2.1p::GFP; myo-2p::mCherry]* |
| ERT90 | *zip-1(jy13)* |
| GL302 | *cde-1(rf34)* |
| IG10 | *tol-1(nr2033)* |
| IG685 | *tir-1(tm3036)* |
| JU1264 | *Caenorhabditis briggsae*, wild strain |
| JU2508 | *drh-1(ok3495)* |
| JU2624 | *[myo-2p::mCherry::unc54; lys-3p::eGFP::tbb2]* II  in *C. elegans* wild strain JU1580 background |
| JU4289 | *agsl219[sysm-1p::GFP + ttx-3p::GFP]* III |
| KU25 | *pmk-1(km25)* |
| MCP553 | *drh-1(mcp553)* |
| N2 | wild-type reference |
| SX2755 | *sdz-6p::GFP; myo-2::mCherry* |
| WM27 | *rde-1(ne219)* |
| ZD101 | *tir-1(qd4)* |
