# Supplementary material for: Naturally-associated bacteria modulate Orsay virus infection of Caenorhabditis elegans
Source: PLoS Pathog. 2024 Jan 17;20(1):e1011947. doi: 10.1371/journal.ppat.1011947 (PMC10824439; doi:10.1371/journal.ppat.1011947)
Supplement: Attachment — Submitted filename: Gonzalez and Felix review.pdf [file ppat.1011947.s012.pdf]

**Natural monobacterial environments modulate viral infection in *Caenorhabditis elegans***

**Summary.**

Host-microbial interactions can modulate host susceptibility to viral infections, but how this occurs is poorly understood. In the current study, the authors screen 67 natural bacterial strains for changes in *Caenorhabditis elegans* response to Orsay viral (OV) infection. Monocultures of most bacterial isolates reduce viral infection, which the authors claim is not due to viral degradation or poor nutrition. Surprisingly, *C. elegans* mutations known to compromise host antiviral responses are dispensable for the increased resistance.

**Overall analysis.**

Unraveling the complex interactions between host, viruses, nutrition, and microbiomes is critical for the development of novel treatments to improve human health. *C. elegans* is a powerful system to reveal these interactions; the authors should be commended for undertaking a large scale analysis of a diverse set of natural bacterial strains. However, there are several major concerns that severely limit enthusiasm for publishing the manuscript in the current form. First, the study is completely descriptive and provides little to no biological insight. There are interesting observations with insufficient follow-up; for instance, titrating in small amounts of natural bacteria acts dominantly to suppress *pals-5p::GFP* induction (a readout of host response to OV). Second, as all of the genetic analysis produced negative results, no putative mechanism is provided for why any bacterial food source would alter *C. elegans* host response to OV infection. However, intuitively the manuscript may be one discovery away from providing the necessary mechanistic insight necessary to warrant further consideration for PLOS Pathogens (suggestions below). Third, some of the experimental approaches are superficial and require additional rigor. For example, throughout the authors use a population assay for activation of *pals-5p::GFP* expression as a binary readout, which may lack sufficient nuance to adequately reveal relevant biology. Additional major but more easily addressable concerns include: 1. the authors overstate several conclusions based on indirect evidence, make a jump in logic, or commit a logical fallacy. Threads started within several supplemental figures need additional rigor and moved into the main text. Lastly, details are missing or difficult to infer in places and the quality of parts of several figures (embedded text) is poor. These and other concerns are described in additional detail below.

**Major Concerns/Considerations.**

1. The study is *completely* descriptive, which diminishes impact because insufficient biological insight is provided and there is no mechanism. Many paragraphs are essentially lists describing how each phenotype is affected by a particular natural bacteria.

2. Analysis is superficial in places. For example, while a 16S phylogenic analysis organizes the natural bacterial strains and reveals some clusters of bacterial strains

that share a similar phenotype (e.g., *Comamonas* result in greater *pals-5::GFP* expression within a population), there is insufficient follow-up into what this means. For example:

a. Are any of the bacteria natural *C. elegans* pathogens (e.g., I thought *Pseudomonas* is a pathogen)? Do any limit survival/lifespan when maintained?
b. Which are gram positive or negative?
c. Are the bacteria rods or spheres?
d. How big are the bacteria (can they pass through the pharyngeal grinder)?
e. Do they colonize the intestinal lumen (opportunistic pathogen)?
f. What is the nutritional content of the bacteria? For example, some gram positive bacteria have thick cell wall polysaccharides covalently bound to peptidoglycan, which would increase relative sugar content.

3. There are several interesting observations that should inform a testable hypothesis but are not adequately explored or developed. Some examples of where the authors could focus in greater depth:
    a. Growth on a natural bacterial results in loss of *pals-5::GFP* induction after OV infection but not after heat stress. This seems like a key observation! What does this mean? A trivial explanation would be that OV fails to adequately enter the host, which could be more rigorously tested using existing transgenic *C. elegans* strains that have the OV genome integrated under the control of an inducible reporter. Assuming OV entry is not impaired this suggests a specific adaptive response.
    b. Titrating in small amounts of natural bacteria suppresses *pals-5p::GFP* induction and requires live-bacteria, which is somewhat surprising as the chosen bacteria are phylogenetically diverse. Nevertheless, this suggests a dominant effect that deserves additional consideration. Do some clades of natural bacteria secrete a dominant factor (which could be heat-labile) while others require ingestion? What happens if you mix natural bacteria that either enhance or limit *pals-5::GFP* expression? If you transfer worms grown in the presence of a natural bacteria, can it horizontally transfer resistance (suggests a secreted response between animals). I'm not sure of the best course, but the dominant effect seems like a key observation.

4. The authors use a population assay for *pals-5::GFP* expression as a binary readout, which may lack sufficient nuance to adequately reveal relevant biology and is too superficial. Additional level of secondary analysis is required (rigor). Does the absolute levels of GFP expression change? Which cells induce *pals-5::GFP?* Representative images *must* be included throughout. Throughout the authors describe the population assays as "similar levels of activation", which is misleading.

5. The authors must more rigorously test whether a bacterial innate immune response is triggering an adaptive response to limit OV. Whether natural bacteria are inducing a bacterial innate immune response is underdeveloped and only superficially examined. Bacterial pathogens are diverse, induce specific reporters, and have unique signaling mechanisms (and genetic requirements).

6. Generally, animals are first introduced to natural bacteria at the same time OV is applied. The authors have not sufficiently distinguished whether exposure to a natural bacteria is inducing an acute adaptive response that is altering host-OV interaction, response, or dynamics of infection. Or whether a natural bacteria is generally protective. For example, if animals are maintained on a natural bacteria (that generates no bacterial innate immune response) as a food source for several generations, would they still be resistant to OV? If not, this suggests that switching food sources results in an adaptive adjustment period; perhaps a temporary alteration in metabolic flux as animals acclimate to the new food source. If animals are moved from a natural bacteria back to OP50, do animals retain resistance to OV? If so, for how many generations?

7. The authors describe horizontal transmission of OV (lines 158-163), but assess vertical transmission (Figure 3)? The text is misleading as horizontal expression isn't actually tested. How far are into the infection are the L4 animals on OP50 just prior to infection? Were they *pals-5::GFP* positive? Were the animals chosen (in any capacity) for a similar intensity of GFP fluorescence (how is this normalized)? It is unclear from the schematic, *exactly* what the authors are doing and neither the figure legend nor method section contain the specific experimental details. This is problematic throughout and other experiments lack adequate description of the methods: for example, line 148: total brood sizes. How was this measured? How many animals? Details are missing.

8. Supplemental Figure 4 should be in the main text and be expanded to include a more rigorous analysis. For example, do these bacterial strains affect the growth rate of *C. elegans,* especially for the bacterial strains that repress *pals-5::GFP* induction? If the proportion of animals with *pals-5* induction as readout, the authors need to confirm that animals fed with different bacterial are exactly at the same stage when infected.

9. It is unclear whether there are acute behavior changes after exposing animals to a new natural bacteria. Does pumping rate change immediately upon transfer to a new food source? Does feeding behavior change? Do animals avoid the bacterial lawn? It has previously been shown that some mutant *C. elegans* with apparent improved bacterial innate immunity were actually better able to sense and avoid the bacterial pathogen; these strains failed to show improved survival when a pathogenic bacteria lawn was spread across the entire plate. Did the authors uniformly coat the plate each bacteria? And was OV also distributed across the full plate? If this was not considered, then reduced *pals-5::GFP* expression within a *C. elegans* population after exposure to a mildly pathogenic bacteria could be explained trivially due to avoiding the lawn. The authors find that animals with reduced *pals-5::GFP* expression after grown on some natural bacteria results in extended reproductive spans, which would be consistent with this possibility. Note, the authors do find one natural bacteria that increases *pals-5::GFP* expression also increases reproductive span; this does not reject the hypothesis that animals with lower *pals-5::GFP* are avoiding another natural bacteria.

10. The authors over-interpret their results to draw conclusions that are too strong. Experiments do not always test what the authors conclude, and the authors tend to draw conclusions from indirect observations. For example, lines 135-138: "*In conclusion, the Acinetobacter BIGb0102 environment enhances infection…*" is an overstatement without quantification of endogenous *pals-5* and RNA1/2 levels (RT-qPCR for each). Again, lines 154-155: "*We thus conclude that the bacterial environments that enable strong viral infection did not do so by generally weakening the host."* is an overstatement as it is unknown whether viral infection is occurring at the same levels and "weakening" lacks informational content.

    Other examples were also found and the authors should be more conservative in their conclusions. For example, line 184: "… *can alter transcriptional response*…" is a jump in logic that is not been tested experimentally. Line 208 subheading: "*Unknown antiviral pathways are involved*…" and line 290-91 "…*unknown antiviral mechanisms*…" are not supported with experimental results and is an "Appeal to Ignorance" logical fallacy (i.e., the absence of evidence is not evidence of absence).

11. Overall, the writing needs to be improved. Examples:
    a. Some of the phrasing is awkwardly constructed: for example, line 32: "*plays a key role in shaping various of its traits*".
    b. In places the authors need to be specific, e.g., line 145: "*to those suppressing it*", line 267: "*certain bacterial environments"* are vague and needs better clarity.
    c. In other places the incorrect tense is used.
    d. The authors should not refer to their prior work in 3$^{rd}$ person (e.g., line 141: *Reported by Frezal*).
    e. There is inconsistent application of the same words (e.g., lines 259-260) strains of bacteria, natural strains of worms.

12. Figure legends for Figures 3-6 fail to describe number of tested animals, trials, statistical analysis, and significance. Links to primary data tables (supplementary files) are missing. Figure legend 2e indicates that 100 animals were assessed for total number of viable progeny with each column? Is this correct?

13. Line 272-275: "*In C. elegans' bacterial environments there is no distinction between food (nutrition) and biotic environment. This overlap is likely significant, given that the lipid content of the nematodes plays a crucial role in viral infections*". I absolutely agree! The authors should assess whether any of the bacterial clades alter major lipid stores, many straightforward assays are routinely used.

14. Heat killed bacteria are not a good food source and could confound results. UV-killed are a good alternative (or perhaps treated with antibiotics in some instances) to confirm results.

15. Was bacterial density normalized between natural bacterial strains? How well do each grow? Many bacteria do not grow as well in LB as *E. coli.*

16. In many figures the text has been rendered in a manner that makes it illegible.

17. Figure 6g is not mentioned in the text?

**Minor Concerns.**

1. Line 169: Figure 7B should be 3B. Line 242: 7G should be 6F. Line 245: Supplementary Figure 7 should be 6. The authors should double check Figure numbers throughout.

2. The definition of “natural” bacteria should be better defined. It seems odd to call *E. coli* “non-natural” bacteria (line 257).

3. Link to the full strain list (Table S2) needs to be mentioned in the first subsection of the Material and Methods.

4. Line 304: “*It was created*…” suggests a new strain was generated. Details on how to validate genotype (*et cetera*) are missing.

5. Line 340: “Evaluation of viral infection” should mention FISH.

6. S4 uses a colorblind unfriendly palette.

7. Fig. 1 use of terms enhancer and suppressor are not strictly known and inaccurate.

8. “Worms” is jargon and has an inherent negative connotation that diminishes the impact of *C. elegans* research; nematodes, *C. elegans,* or animals are better descriptors.
