# Supplementary material for: Naturally-associated bacteria modulate Orsay virus infection of Caenorhabditis elegans
Source: PLoS Pathog. 2024 Jan 17;20(1):e1011947. doi: 10.1371/journal.ppat.1011947 (PMC10824439; doi:10.1371/journal.ppat.1011947)
Supplement: Attachment — Submitted filename: Response_reviewers_PLOS Pathogens_Gonzalez and Felix 2023.pdf [file ppat.1011947.s013.pdf]

Dear González,

Thank you very much for submitting your manuscript "Natural monobacterial environments modulate viral infection in Caenorhabditis elegans" for consideration at PLOS Pathogens. As with all papers reviewed by the journal, your manuscript was reviewed by members of the editorial board and by several independent reviewers. In light of the reviews (below this email), we would like to invite the resubmission of a significantly-revised version that takes into account the reviewers' comments. As you can see, several reviewers requested more information about the bacteria tested in this study. In particular it would be important to see more information about how these bacteria affect ingestion of virus in C. elegans, as effects here could have a significant impact on infection. Also important would be to show more quantitative and direct measurements of viral infection, particularly in places where conclusions are based on GFP reporter expression only, as an indirect read-out of infection.

We cannot make any decision about publication until we have seen the revised manuscript and your response to the reviewers' comments. Your revised manuscript is also likely to be sent to reviewers for further evaluation.

When you are ready to resubmit, please upload the following:
[1] A letter containing a detailed list of your responses to the review comments and a description of the changes you have made in the manuscript. Please note while forming your response, if your article is accepted, you may have the opportunity to make the peer review history publicly available. The record will include editor decision letters (with reviews) and your responses to reviewer comments. If eligible, we will contact you to opt in or out.
[2] Two versions of the revised manuscript: one with either highlights or tracked changes denoting where the text has been changed; the other a clean version (uploaded as the manuscript file).

Important additional instructions are given below your reviewer comments.
Please prepare and submit your revised manuscript within 60 days. If you anticipate any delay, please let us know the expected resubmission date by replying to this email. Please note that revised manuscripts received after the 60-day due date may require evaluation and peer review similar to newly submitted manuscripts.

Thank you again for your submission. We hope that our editorial process has been constructive so far, and we welcome your feedback at any time. Please don't hesitate to contact us if you have any questions or comments.

Dear Editorial Team and Reviewers,

Thank you for the comprehensive review and constructive feedback on our manuscript. We truly value the time and expertise provided by the reviewers in evaluating our work. We have addressed their concerns to the best of our abilities. In response to the suggestions, we have undertaken significant revisions and performed additional experiments. As outlined below, in this revision we:

- Repeated the experiment involving viral persistence over generations, using FISH, confirming the reduced proportion of infected animals on the resistance-inducing bacteria.
- Quantified the viral load using RT-qPCR across various bacterial environments confirming the reduction in viral load on the resistance-inducing bacteria.
- [additional experiment, not as a response to reviews] Tested the resistance-inducing bacteria on the pathosystem *Caenorhabditis briggsae* – Santeuil virus and observed a specific effect on *C. elegans* – Orsay virus.
- [additional experiment, not as a response to reviews] Expanded the strain tested to include species belonging to *Bacillota*.
- Verified the identity of the key natural bacteria studied and corrected experiments performed with wrongly identified bacteria (the two *Leucobacter luti*).
- Examined avoidance behaviors and conducted tests over bacterial lawns covering the entire plate, demonstrating that bacterial lawn avoidance is not the cause of the resistance.
- Measured the bacteria's impact on *C. elegans* developmental rates and tested mixed environments that restore the developmental rate, with results showing that the hypothesis of bacterial suppression through this mechanism did not hold.
- Investigated the effect of the bacterial supernatant, showing that the viral resistance is probably not caused by a bacterial secreted factor.
- Incorporated images to display the *pals-5:GFP* reporter.
- Incorporated images of an antibacterial immune response reporter on the studied bacterial environments.

We would like to point out that for the experiment depicted in Figure 6B, all animals were initially exposed to the virus in the uniform *E. coli* OP50 environment. After this exposure, they were divided into subpopulations and moved to different bacterial environments without virus. Given this methodology, all animals would have acquired the virus at a similar rate in the OP50 setting. The subsequent transfer to alternate bacterial environments is what induced resistance to the infection, thus allowing us to discard the hypotheses of the second bacterial environment hindering viral entry from the plate into the gut lumen (including via slower pumping rates).

Furthermore, we have refined the manuscript to enhance clarity and have made substantial improvements to the figure legends. We regret the oversight in the presentation of the figures in our initial submission and have ensured that they are correctly rendered in this version. Please note that we have used the PACE tool suggested by PLOS to transform the figures into the appropriate format. However, after the site builds the PDF the images look slightly blurred; the images look properly when they are downloaded (upper right, “Click here to access/download;Figure;Figure X.tif”)

In summary, we believe that the extensive revisions and new data enhance the quality and depth of our manuscript.

Kind regards,
The authors.

PART II – MAJOR ISSUES: KEY EXPERIMENTS REQUIRED FOR ACCEPTANCE

REVIEWER 1

The conclusion in line 158 "Natural bacteria eliminate OrV in infected nematode populations within two generations" is not fully supported by the evidence presented. This conclusion is drawn from an experiment in which worms are infected with OrV on OP50, then transferred to an infection-suppressing bacterial environment and allowed to reproduce. My issue with this conclusion is that the only readout used to assess infection in this experiment was pals-5p::GFP infection reporter readout, which is not a direct readout of viral infection but of worm immune response. The claim of "virus elimination" (lines 158 & 169-170) is therefore not fully supported by this data, which could also be explained by a change in the transcriptional activation of pals-5 in the progeny. The authors should either moderate their language on this point (in the lines noted above), or conduct RNA FISH staining to directly assess viral infection in order to support this claim.

We have repeated this experiment using RNA FISH to provide a more direct evidence of viral presence. The new results, in Figure 6, support our initial claim.

REVIEWER 2

The authors should examine if the key (5) bacterial strains they test throughout their study alter C. elegans feeding/avoidance behaviors.

We have added information regarding avoidance behaviors (Figure 5A). To ensure that avoidance was not the primary reason for the lack of infection, we designed an experiment where both the bacteria and the viral inoculum covered the entire plate. In this scenario where the nematodes could not evade bacteria nor virus, we observed the same absence of infection (Figure 5B). In addition, pumping rates cannot be the reason for the suppression, as we performed an experiment where the animals were initially virally-infected on OP50 then transferred to different bacteria (Figure 6) .

Related to the first point, the authors should address the possibility that the key bacterial strains they investigate negatively impact C. elegans development (compared to OP50). Given that worms at different stages of development may have altered OV susceptibilities, the apparent impact of a bacterial culture on OV infection rates may be an indirect effect of altered growth/development rates. Reduced development rates caused by bacteria could also explain the reduced C. elegans brood sizes observed with most of these bacterial cultures.

The developmental rates across different bacterial cultures were added in the revised version (Figure 3B). We do see variation among the six tested bacteria, however this does not closely match their effect on viral infection. In addition, given that most of the six tested bacteria including the suppressing bacteria tended to slow down developmental rate, we have now included an experiment that shows that mixed environments that restore developmental rates do not restore susceptibility to infection (Figure 4B and Supplementary Figure 6).

The authors should validate key findings with more quantitative methods to assess differences in OV replication (e.g. qPCR of viral RNA to measure viral loads).

We have employed RT-qPCR to quantify the accumulation of viral RNA1 across the six bacterial strains studied in depth (Figure 2D). This shows that the suppressive bacteria strongly inhibit viral infection.

REVIEWER 3

1. The study is completely descriptive, which diminishes impact because insufficient biological insight is provided and there is no mechanism. Many paragraphs are essentially lists describing how each phenotype is affected by a particular natural bacteria.

While the study is primarily descriptive, we believe this approach provides a necessary foundational understanding of the topic.

2. Analysis is superficial in places. For example, while a 16S phylogenic analysis organizes the natural bacterial strains and reveals some clusters of bacterial strains that share a similar phenotype (e.g., Comamonas result in greater pals-5::GFP expression within a population), there is insufficient follow-up into what this means. For example:
a. Are any of the bacteria natural C. elegans pathogens (e.g., I thought Pseudomonas is a pathogen)? Do any limit survival/lifespan when maintained?
Not all *Pseudomonas* are pathogens. For example, *Pseudomona lurida* MYb11, part of *C. elegans* microbiota, is considered non-pathogenic (Dirksen et al., 2020 G3).
Whether a bacterium is considered pathogenic depends on the definition of pathogenicity and the reference used for comparison. Post-reproductive adult lifespan is not ecologically relevant during exponential population growth. As an example, *P. lurida* MYb11 increases offspring production (in comparison to OP50) but reduces lifespan (in comparison to OP50) (Kissoyan et al., 2022 Front. Cell. Infect. Microbiol). Hence, depending on the phenotype considered (and the reference for comparison), *P. lurida* MYb11 could be considered pathogenic or not.

b. Which are gram positive or negative?
This information has been included in the text: *"In samples taken from Europe (specifically France and Spain), it was found that the most abundant phylum was Pseudomonadota (synonym: Proteobacteria, a group of Gram-negative bacteria). Other phyla such as Bacteroidota (synonym: Bacteroidetes, Gram-negative bacteria), Bacillota (synonym: Firmicutes, Gram-positive bacteria), and Actinomycetota (synonym: Actinobacteria, Gram-positive bacteria) were also identified"*.

c. Are the bacteria rods or spheres?
d. How big are the bacteria (can they pass through the pharyngeal grinder)?
e. Do they colonize the intestinal lumen (opportunistic pathogen)?
For the bacteria we studied in depth, in young adults, only *Lelliottia* JUb276 clearly colonizes the intestinal lumen of ERT54 (see pictures below).

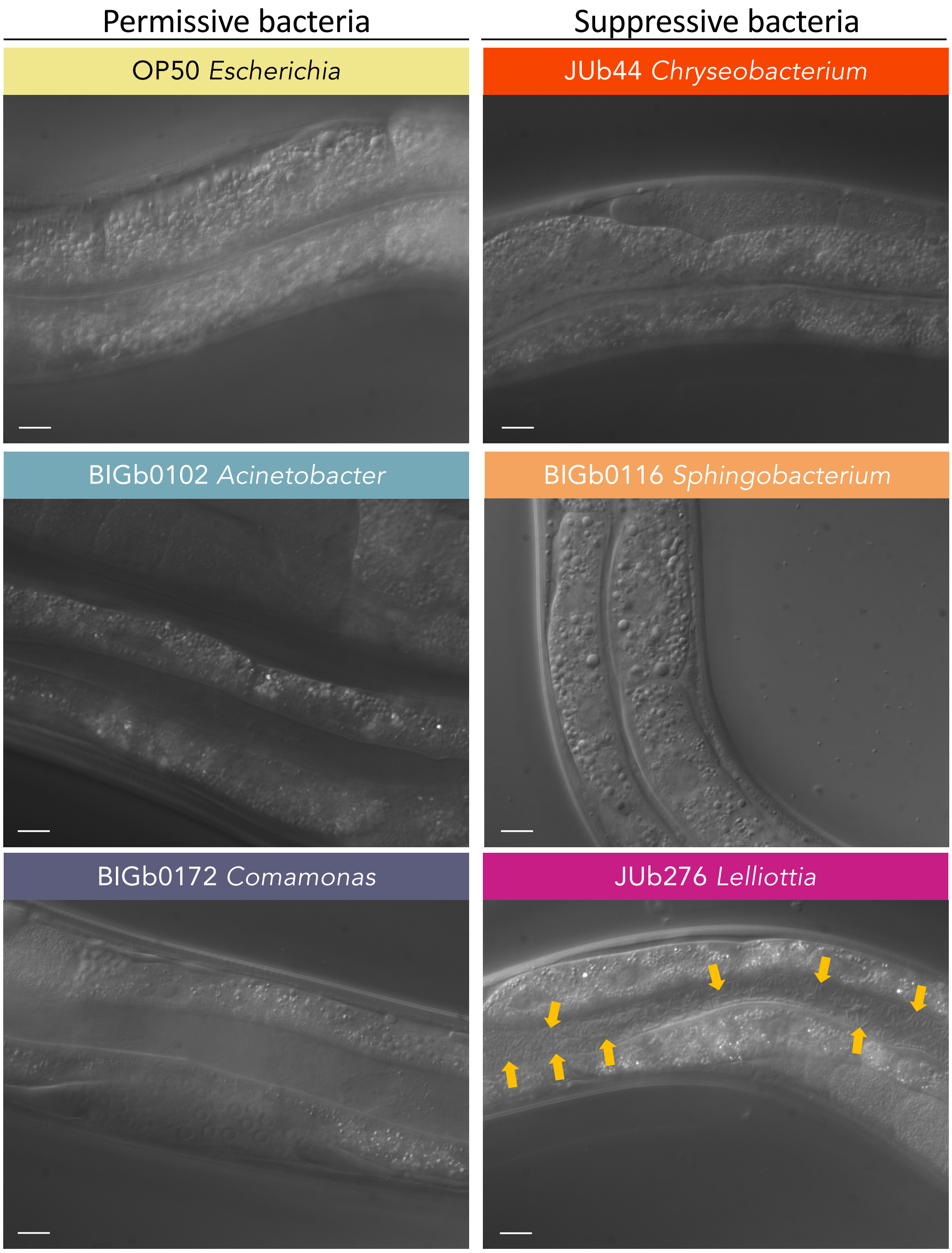

Intestinal lumen of 3-day-old ERT54 animals grown on different bacterial environments was observed with Nomarski optics with a 100× objective mounted on a Zeiss AxioSKOP microscope and captured with a CoolSNAP ES (Photometrics) camera. Yellow arrows point to live bacteria. Scale bar represents 10 microns.

f. What is the nutritional content of the bacteria? For example, some gram positive bacteria have thick cell wall polysaccharides covalently bound to peptidoglycan, which would increase relative sugar content.

We lack information about the nutritional content of the bacteria.

3. There are several interesting observations that should inform a testable hypothesis but are not adequately explored or developed. Some examples of where the authors could focus in greater depth:
a. Growth on a natural bacterial results in loss of pals-5::GFP induction after OV infection but not after heat stress. This seems like a key observation! What does this mean? A trivial explanation would be that OV fails to adequately enter the host, which could be more rigorously tested using existing transgenic C. elegans strains that have the OV genome integrated under the control of an inducible reporter. Assuming OV entry is not impaired this suggests a specific adaptive response.

We distinguish below between entering 1) the intestinal lumen and 2) the intestinal cells.

1) Regarding entry into the intestinal lumen:

The bacteria do not suppress by preventing OV from entering the intestinal lumen: In the experiment of Figure 6B, we enable virus entry in a bacterial environment where the animals are susceptible to infection (OP50) and then transfer them to virus-free natural bacteria. The infection was established in some environments, providing a positive control for viral entry. It was repressed in other environments, in which the resistance could thus not be caused by impairment of virus entry.

2) Regarding entry into the cells:

We used the strain from David Wang's laboratory carrying the OrV genome integrated under the control of an inducible reporter:

WUM29	{*virIs1[PHIP::***OrsayRNA1***;PHIP::***OrsayRNA2***; Pmyo-2::YFP]; jyIs8[Ppals-5::GFP; Pmyo-2::mCherry]; rde-1(ne219)* V}

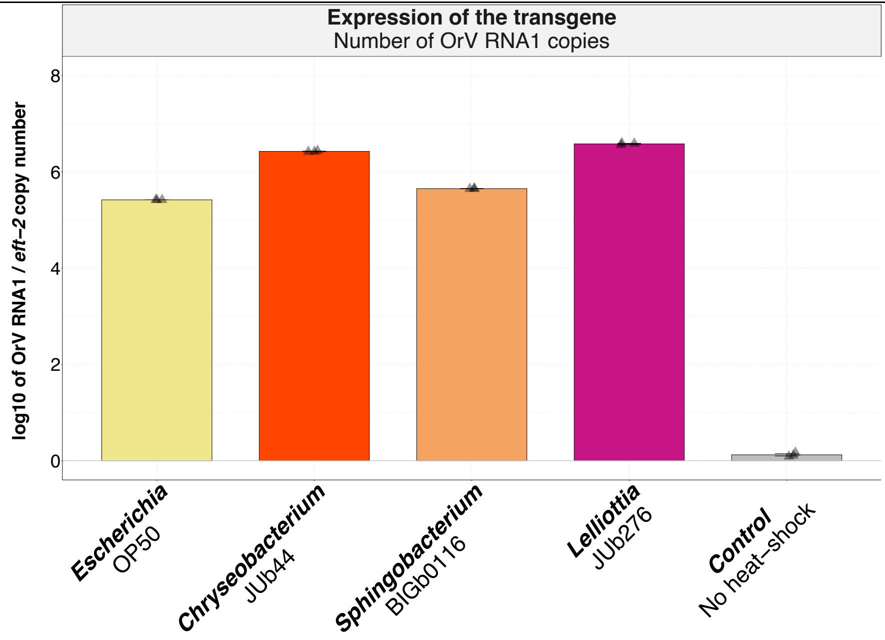

Axenic embryos of strain WUM29 carrying an endogenous transgene containing the OrV genome under the control of a heat-inducible promoter (Jiang et al., 2014, Jiang et al., 2017) were transferred to a lawn of the indicated bacterial strain. Three days later the young adults were heat-shocked at 33°C for 2 h. After the heat-shock the animals were transferred at 20°C for 24 h and collected for measuring their viral load: number of RNA1 copies, normalized to *eft-2*. Each data point represents one of three technical replicates of the RT-qPCR performed on a pool of three independent populations, each one of ca 200 animals.

This result seems to indicate that the replication cycle is not affected by the bacteria (at least in the *rde-1* background). However, we do not see this result as strong enough to be included in the manuscript, because: (i) we cannot rule out that the detected RNA is a transcriptional product from the earlier induction of the promoter rather than a replication product of the viral machinery; (ii) we cannot rule out that the bacteria activate the promoter of the transgene; (iii) even assuming that the replication is not affected, we could not conclude that the repression is happening at virus entry into the cell, as it could also target other processes before replication.

For these reasons, we do not feel confident to include this information in the manuscript.

b. Titrating in small amounts of natural bacteria suppresses pals-5p::GFP induction and requires live-bacteria, which is somewhat surprising as the chosen bacteria are phylogenetically diverse. Nevertheless, this suggests a dominant effect that deserves additional consideration. Do some clades of natural bacteria secrete a dominant factor (which could be heat-labile) while others require ingestion?
We included an experiment to test whether the bacterial supernatant (Figure 7F) could induce resistance; it did not, so the resistance does not seem to be induced by a secreted factor.

What happens if you mix natural bacteria that either enhance or limit pals-5::GFP expression?
To answer this, we included a new experiment where a mix of 20% enhancer bacteria (*Actinobacteria* BIGb0102) and 80% suppressor bacteria was seeded into the plates (Figure 7B). Resistance was still observed, confirming the dominant effect of suppressing bacteria as observed on *E. coli* OP50.

We cannot control growth and interactions between live bacteria once on the plate. To minimize this, we find more accurate to add a bacterial culture on top of a plate already seeded with a different bacterium. This is tested in the supplementation experiment performed in Figure 7B.

If you transfer worms grown in the presence of a natural bacteria, can it horizontally transfer resistance (suggests a secreted response between animals). I'm not sure of the best course, but the dominant effect seems like a key observation.
We appreciate this good suggestion but we find it complicated to transfer the animals in a way that we are sure that the associated bacterium is not being transferred too.

4. The authors use a population assay for pals-5::GFP expression as a binary readout, which may lack sufficient nuance to adequately reveal relevant biology and is too superficial. Additional level of secondary analysis is required (rigor). Does the absolute levels of GFP expression change? Which cells induce pals-5::GFP? Representative images must be included throughout. Throughout the authors describe the population assays as "similar levels of activation", which is misleading.
We have now quantified the viral load by RT-qPCR (e.g. Figure 2D). Concerning the *pals-5* reporter, the text has been reworded to be clearer. We used the reporter activation as a straightforward quantitative method to infer the infection status of the population of animals (i.e. proportion of infected vs non-infected). Images of *pals-5::GFP* are now incorporated (Supplementary Figure 2). The reporter is activated in intestinal cells.

5. The authors must more rigorously test whether a bacterial innate immune response is triggering an adaptive response to limit OV. Whether natural bacteria are inducing a bacterial innate immune response is underdeveloped and only superficially examined. Bacterial pathogens are diverse, induce specific reporters, and have unique signaling mechanisms (and genetic requirements).

A comprehensive study of the possible impact of *C. elegans* immune responses against bacteria on virus infection is beyond the scope of this manuscript. However, we tested a major antibacterial pathway, the p38/MAPK pathway, using mutants (Supplementary Fig 7A&B). In addition, we now include images of animals carrying a *sysm-1* reporter exposed to all five selected naturally-associated bacteria. None of these bacteria activated the reporter (Supplementary Fig 7C).

6. Generally, animals are first introduced to natural bacteria at the same time OV is applied. The authors have not sufficiently distinguished whether exposure to a natural bacteria is inducing an acute adaptive response that is altering host-OV interaction, response, or dynamics of infection. Or whether a natural bacteria is generally protective. For example, if animals are maintained on a natural bacteria (that generates no bacterial innate immune response) as a food source for several generations, would they still be resistant to OV? If not, this suggests that switching food sources results in an adaptive adjustment period; perhaps a temporary alteration in metabolic flux as animals acclimate to the new food source. If animals are moved from a natural bacteria back to OP50, do animals retain resistance to OV? If so, for how many generations?

As stated, the experiment in Figure 6B tests introducing OV prior to exposure to the natural bacteria.

We performed the suggested converse experiment of exposing *C. elegans* to natural bacteria for several generations then to OV, with the same effect. Transient alteration in the metabolic flux does not seem to be the cause of the observed resistance. (We did not include this experiment in the manuscript.)

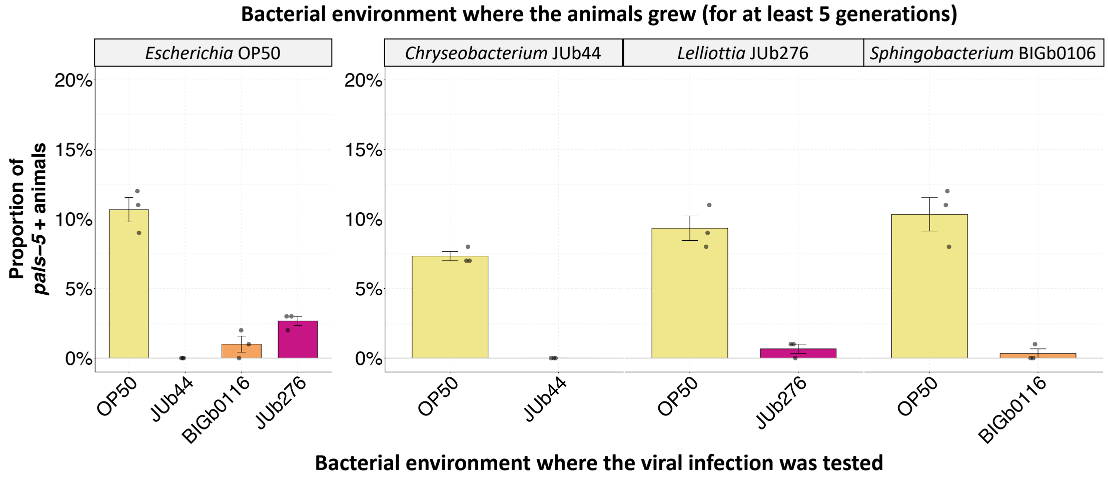

7. The authors describe horizontal transmission of OV (lines 158-163), but assess vertical transmission (Figure 3)? The text is misleading as horizontal expression isn't actually tested. How far are into the infection are the L4 animals on OP50 just prior to infection? Were they pals-5::GFP positive? Were the animals chosen (in any capacity) for a similar intensity of GFP fluorescence (how is this normalized)? It is unclear from the schematic, exactly what the authors are doing and neither the figure legend nor method section contain the specific experimental details. This is problematic throughout and other experiments lack adequate description of the methods: for example, line 148: total brood sizes. How was this measured? How many animals? Details are missing.

There is no vertical transmission of OrV. We now clarified the text to remove the ambiguity: *"In natural environments, viral infections spread within populations as infected organisms continuously produce and release viruses (41). In order to create conditions where horizontal virus transmission must occur for the viral infection to be maintained, we transferred individuals*

from an infected C. elegans population, previously inoculated on Escherichia OP50, to the resistance-inducing bacterial environments"

We have extended the method description to be more informative. For example, in the legend of the total brood size now it is detailed *"Figure 3. Impact of selected bacteria on the fitness and developmental rates of C. elegans. (A) Upper panel shows total brood size of non-infected ERT54 animals when placed on each bacterial strain. Lower panels represent the daily production of viable progeny of non-infected ERT54 animals. Two separate experiments were conducted, in which the viable progeny of individual animals was monitored daily, with at least 5 individuals observed per bacterial type in each experiment. The upper panel represents the total progeny of those shown in the lower panels; the two experiments are represented by triangles and circles, respectively"*. The Methods has been expanded with new sections: Mixed bacterial environment experiments, OrV persistence over generations experiments, Coincubation of bacterial culture and virus inoculum, Common garden inoculation experiment.

8. Supplemental Figure 4 should be in the main text and be expanded to include a more rigorous analysis. For example, do these bacterial strains affect the growth rate of C. elegans, especially for the bacterial strains that repress pals-5::GFP induction? If the proportion of animals with pals-5 induction as readout, the authors need to confirm that animals fed with different bacterial are exactly at the same stage when infected.

Impact of the bacteria on development is now included (Fig 3B).

The virus is added to the plate on embryos (which cannot be infected). They may start to acquire the virus from the L1 stage. All larval stages of *C. elegans* are susceptible to OrV infection. In addition, we have included an experiment that measures by FISH the susceptibility of animals grown in a bacterial mixture, where animals show the same developmental rate than in a susceptible bacterial environment and they are not infected by the virus (Figure 4B and Supplementary Figure 6). This experiment confirms that the developmental rate is not the cause of the observed resistance - although we cannot rule out that it subtly alters quantitatively the infection.

9. It is unclear whether there are acute behavior changes after exposing animals to a new natural bacteria. Does pumping rate change immediately upon transfer to a new food source? Does feeding behavior change? Do animals avoid the bacterial lawn? It has previously been shown that some mutant C. elegans with apparent improved bacterial innate immunity were actually better able to sense and avoid the bacterial pathogen; these strains failed to show improved survival when a pathogenic bacteria lawn was spread across the entire plate. Did the authors uniformly coat the plate each bacteria? And was OV also distributed across the full plate? If this was not considered, then reduced pals-5::GFP expression within a C. elegans population after exposure to a mildly pathogenic bacteria could be explained trivially due to avoiding the lawn. The authors find that animals with reduced pals-5::GFP expression after grown on some natural bacteria results in extended reproductive spans, which would be consistent with this possibility. Note, the authors do find one natural bacteria that increases pals-5::GFP expression also increases reproductive span; this does not reject the hypothesis that animals with lower pals-5::GFP are avoiding another natural bacteria.

We now describe the level of bacterial avoidance. We did not observed avoidance during the first 48 hours. After this time we observed some aversion in one suppressive bacteria (JUb276) and in one permissive one (BIGb102). Hence, aversion to the bacteria did not match their effect on viral infection (Figure 5A).

We also tested the impact of the bacteria when they cover the full plate and observed the same results as in the standard bacterial lawn (Figure 5B).

10. The authors over-interpret their results to draw conclusions that are too strong. Experiments do not always test what the authors conclude, and the authors tend to draw conclusions from

indirect observations. For example, lines 135-138: "In conclusion, the Acinetobacter BIGb0102 environment enhances infection…" is an overstatement without quantification of endogenous pals-5 and RNA1/2 levels (RT-qPCR for each). Again, lines 154-155: "We thus conclude that the bacterial environments that enable strong viral infection did not do so by generally weakening the host." is an overstatement as it is unknown whether viral infection is occurring at the same levels and "weakening" lacks informational content.

We believe that that the reviewer considers infection at the individual level while we measure it as the susceptibility at the population level. We now explain in the text that throughout the manuscript, if a bacterial environment increases the proportion of infected animals in a population, we express the fact in saying that this environment enhances viral incidence.

In addition, we now measured viral load by RT-qPCR of the virus. The results are fully concordant with those on the proportion of *pals-5::GFP* individuals.

The "weakening" statement was indeed imprecise and has been removed.

We did not quantify *pals-5* by RT-qPCR. If the reviewer is worried about general effects of the bacteria or virus on repeated transgene expression, the strain contains a constitutive *myo-2::Red* marker that was not silenced in the pharynx by suppressive bacteria.

Other examples were also found and the authors should be more conservative in their conclusions. For example, line 184: "… can alter transcriptional response…" is a jump in logic that is not been tested experimentally. Line 208 subheading: "Unknown antiviral pathways are involved…" and line 290-91 "…unknown antiviral mechanisms…" are not supported with experimental results and is an "Appeal to Ignorance" logical fallacy (i.e., the absence of evidence is not evidence of absence).

We tried to tone down our statements. For example, the section *"Unknown antiviral pathways are involved in the bacterial suppression of viral infection"* was renamed *"Intact host antiviral pathways are not required for bacterial suppression of viral infection"*. The mentions of an alteration of the transcriptional response have been replaced by *"alteration of the pals-5p::GFP activation"*. We also now mention: *"It is also possible that rather than through DRH-1 mediated activation of antiviral pathways, the suppression operates because viral RNA does not enter the intestinal cells on suppressive bacteria, or that the cells are not competent for some part of the lifecycle of the virus, such as replication, translation or packaging."*

11. Overall, the writing needs to be improved. Examples:

a. Some of the phrasing is awkwardly constructed: for example, line 32: "plays a key role in shaping various of its traits".

Phrasing has been revised in the whole manuscript. The highlighted sentence has been changed to *"The biotic environment of an organism influences many of its traits".*

b. In places the authors need to be specific, e.g., line 145: "to those suppressing it", line 267: "certain bacterial environments" are vague and needs better clarity.

We worked in improving the clarity on the whole paper. In the specific cases mentioned we have changed:

- *"to those suppressing it"* has been removed.
- *"certain bacterial environments"* to *"We found that BIGb0116 and JUb276 bacterial strains may confer protection against viral infections while simultaneously reducing host fitness".*

c. In other places the incorrect tense is used.

Revised.

d. The authors should not refer to their prior work in 3rd person (e.g., line 141: Reported by Frezal).

Changed *"We tested JUv2572, an OrV strain reported by Frézal et al. (39) to be…"* to *"We tested JUv2572, an OrV strain reported to be more infectious than JUv1580 and prone to*

infecting more anterior host intestinal cells (39)".
e. There is inconsistent application of the same words (e.g., lines 259-260) strains of bacteria, natural strains of worms.
The text has been revised to state clearly if with the word strain we are referring to the nematodes, the bacteria or the virus.

12. Figure legends for Figures 3-6 fail to describe number of tested animals, trials, statistical analysis, and significance. Links to primary data tables (supplementary files) are missing. Figure legend 2e indicates that 100 animals were assessed for total number of viable progeny with each column? Is this correct?
This information has been systematically added in the Figure legends. The methods for statistical analysis are detailed in the method section and the test used for each panel mentioned in the corresponding legend of the figure.

Supplementary files were uploaded as Supporting Information Item to PLOS Pathogens; at the end of the document you received to review there should be two pages, each one with a link to each supplementary file:

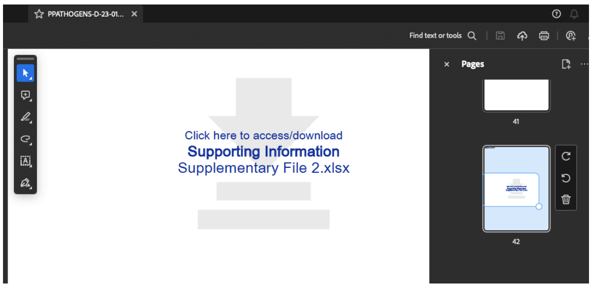

13. Line 272-275: "In C. elegans' bacterial environments there is no distinction between food (nutrition) and biotic environment. This overlap is likely significant, given that the lipid content of the nematodes plays a crucial role in viral infections". I absolutely agree! The authors should assess whether any of the bacterial clades alter major lipid stores, many straightforward assays are routinely used.
JUb276 does decrease lipid store content compared to other bacteria. However, we observed similar lipid store content of young adults in other bacteria, whether enhancing or suppressing (see images below). Thus, the altered lipid stores could in principle be a specific mechanism for JUb276 but does not explain the suppression by other bacteria.

The supplementation experiments shown in Figure 7 disprove the hypothesis that the bacteria suppress by not providing sufficient nutrients.

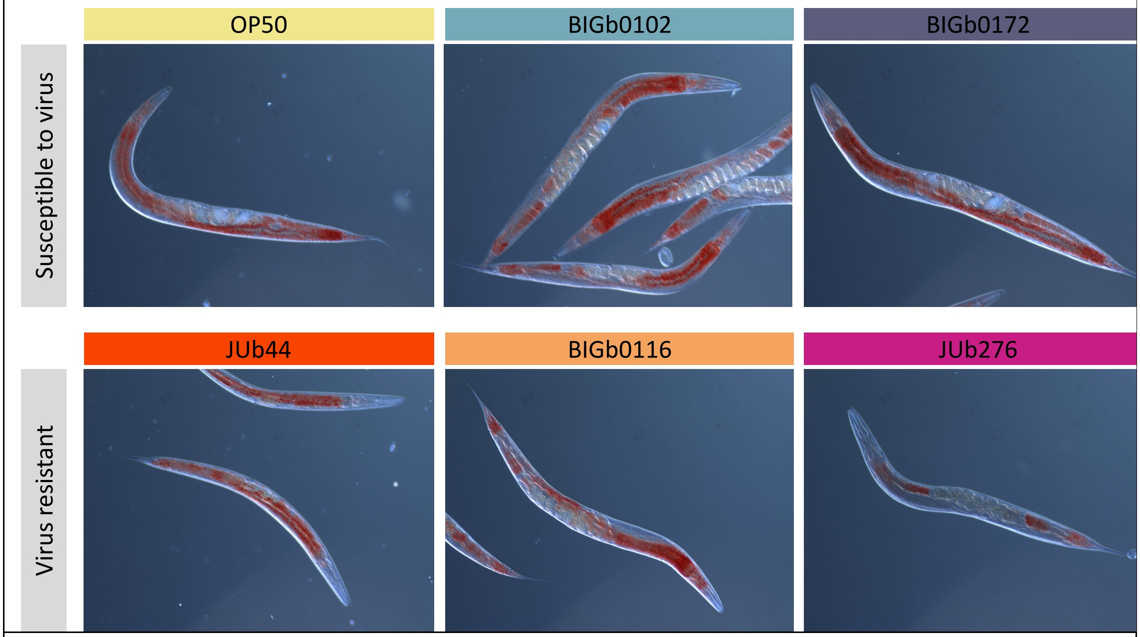

We stained ERT54 animal neutral lipids using Oil Red O (ORO) and following the protocol described elsewhere (Stuhr et al., 2022). Briefly, 3-day-old animals were fixed for 3 minutes, rotating in 60% isopropanol at RT. Then, they were mixed by rotation at RT with 600 µL of 60% ORO staining solution for 2 hours. After the staining, the nematodes were washed with PSB buffer for 30 min while rotating at room temperature. ORO stained animals were visualized using a 10x objective mounted on an AxioSKOP2 (Zeiss) microscope and captured with a Nikon DS-Fi1 digital camera.

14. Heat killed bacteria are not a good food source and could confound results. UV-killed are a good alternative (or perhaps treated with antibiotics in some instances) to confirm results. While we understand the concerns about heat-killed bacteria, in our experiments they were added on top of a non-heat killed bacterial lawn, ensuring that animals were adequately fed. The concern remains that heat may denature a required compound for suppression so we have been careful on the interpretation of this experiment.

15. Was bacterial density normalized between natural bacterial strains? How well do each grow? Many bacteria do not grow as well in LB as E. coli.
Bacterial density of the liquid cultures was not normalized. Note that even if we controlled the initial density of the liquid culture, we do not control how much the bacteria would grow once seeded on the NGM plate.

All naturally-associated bacteria grew well in LB. We measured the number of colony-forming units (CFU) of our selected bacteria in our growth conditions and all grow well: for OP50, BIGb0102, BIGb0116, JUb44, and JUb276 the CFUs are all on the order of 10^{10}, while for BIGb0172 it is on the order of 10^{8}.

16. In many figures the text has been rendered in a manner that makes it illegible. We apologize for that. Figures should be legible in this version. Please note that we have used the PACE tool suggested by PLOS to transform the figures into the appropriate format. However, after the site builds the PDF the images look slightly blurred; the images look properly when they are downloaded (upper right, “Click here to access/download;Figure;Figure X.tif”)

17. Figure 6g is not mentioned in the text?
Corrected now.

PART III – MINOR ISSUES: EDITORIAL AND DATA PRESENTATION MODIFICATIONS

REVIEWER 1

Regarding lines 144-155: It would be beneficial here to clarify that progeny production is only one dimension of worm health; there are known instances of trade-offs between progeny production and other health measures such as lifespan or immunity. It would also help the clarity of this section to note that although the authors find brood size effects in Acinetobacter BIGb0102 (higher viral susceptibility but also higher brood size) and several of the other strains (lower viral susceptibility and also lower brood size) that seem to argue against the general-health hypothesis, they do find one strain that contradicts this pattern. Infection-enhancing strain Comamonas BIGb0172 also has lower brood size, and although they note that the brood size on BIGb0172 is similar to that on several other bacterial strains that protect against viral infection, this does not rule out a general health-suppressive effect at work in the specific case of BIGb0172.

We agree that total progeny production does not cover all *fitness* components; for example the rate (as shown in lower panels in Figure 3A) is also important. We insist that post-reproductive lifespan is not a component of fitness in the growth conditions we use. We do not really know how to quantify *health*. The weakening statement has been removed.

There are issues with the spacing/fonts on nearly all of the figures that make some of the labels difficult to read in the PDF. This appears to be some sort of formatting/encoding issue.

We apologize for the quality of the figures. Figures now should be visualized correctly. Please note that we have used the PACE tool suggested by PLOS to transform the figures into the appropriate format. However, after the site builds the PDF the images look slightly blurred; the images look properly when they are downloaded (upper right, "Click here to access/download;Figure;Figure X.tif")

The clarity of the figure legends could be improved in several places:
-In figure 2, the legend states that "Each data point represents an independent population of ~100 animals"; since individual data points are not shown in the graphs (only means), this is somewhat unclear. The authors should either adjust their language to make it clear whether they mean each experimental replicate used 100 worms (and how many replicates were performed), or alternatively show the individual data points for each replicate in addition to the mean.
-Overall, it is difficult to discern for most experiments shown (except figure 1) how many replicates were performed. This information should be added to the figure legends and the Materials and Methods.

Figure legends have been modified to be more informative and now they include number of replicates performed and number of animals tested per replicate, statistical test performed, and when needed a reference to the corresponding Methods section. This section has been substantially expanded.

As an example, the legend of Figure 4 (Figure 7 in the revised version) was modified from:

"Figure 4. Assessment of potential virion degradation by different bacteria. (A) Activation of an infection reporter in animals on Escherichia OP50, challenged with viruses previously incubated with various bacterial cultures. (B) Animal response to OrV challenge after initial exposure to the virus in Escherichia OP50 and subsequent transfer to different, non-virus-inoculated, bacteria."

to the present version:

"Figure 7. Suppressive bacterial environments act after viral entry. (A) Activation of the pals-5p::GFP reporter in ERT54 animals on Escherichia OP50, challenged with viruses previously incubated with various bacterial cultures (detailed in Methods section "Coincubation of bacterial

*culture and virus inoculum”). Four replicate populations were evaluated per condition. Each data point represents a biological replicate, with at least 100 animals assayed per population. Upper panel shows a schematic representation of the experimental design (detailed in methods). (B) Proportion of GFP-positive ERT54 animals after initial exposure to the virus on Escherichia OP50 and subsequent transfer to different, non-virus-inoculated, bacteria. Upper panel shows a schematic representation of the experimental design (detailed in Methods section “Common garden inoculation experiment”). For the transcriptional response, three replicate populations were evaluated per condition and experiment. These three populations were pooled and the vRNA FISH stained to quantify the proportion of infected animals. Each data point represents a biological replicate, with at least 100 animals assayed per population. Independent experiments performed on different days are represented by the shape of the data point. Data are presented as mean ± standard error. "dpi"= days post-inoculation. Black symbols indicate the significance of the difference between the labeled bacteria and the Escherichia OP50 reference: *** P < 0.001; ** P < 0.01; * 0.01 < P < 0.05; P values higher than 0.05 are labeled as “ns”. Significance was calculated for panel A using an analysis of variance with bacteria as a factor and Dunnett’s test for post hoc analyses and for panel B using a general linear-mixed model where bacteria was the fixed factor with experiments as random effect and Tukey contrasts for post hoc analyses.”*

In several places the text references an incorrect figure:
-on lines 204-205, it should be Supplementary figure 6D not 5D
-on line 232, it should be Supplementary figure 5C not 6D
-on line 233, there is no panel H in figure 6
-on line 242, there is no figure 7
-on line 254, it should be Supplementary Figure 6, not 7
Corrected.

REVIEWER 2

1. There is very little justification for the specific bacterial isolates used other than they are “natural bacterial strains”. Are certain strains/groups more commonly associated with C. elegans in the environment and thus may be more relevant to modulating virus susceptibility in the wild?

Concerning the initial set of bacteria, we attempted to cover the breadth of taxa found in the environment of *C. elegans* by Samuel et al. (reference 15 in the manuscript, figures in this reply belong to this reference). The dominant bacteria were *Proteobacteria*, particularly *Enterobacteriaceae.* We also found bacteria belonging to *Firmicutes*, *Bacteroidetes*, and *Actinobacteria*.

Most of the bacteria tested in our study are *Proteobacteria* (syn. of *Pseudomonata*), and we also tested some *Actinomycetota* (syn. *Actinobacteria*) and *Bacteroidota* (syn. *Bacteroidetes).* In this revised version we have included the test of some new strains to have representation of *Firmicutes*. We made this information clear in the text.

The 5 bacteria studied in depth were randomly selected.

2. The authors should clearly state for each figure (e.g. in figure legends) which specific strain of C. elegans (e.g. ERT54?) was used for each assay.

We have ensured that the nematode strain used is explicitly stated in each figure legend.

3. Supplementary Figure 1-it would be helpful for the authors to comment further on how pals-5 activation was scored, including representative images of negative vs. positive activation

phenotypes as well as control images where no virus was added because the authors indicate that the pals-5 reporter was not activated by any of the screened bacteria in the absence of virus infection but no data are shown to support this statement. Was GFP signal simply scored qualitatively as positive or negative if any part of the animal appeared green or was there some sort of quantitative measurement of GFP signal that had to meet a particular threshold to be scored as positive?
The GFP signal was scored as a binary trait, quantified over the population of animals. The binary trait is conditioned on a threshold of detection in our setting. We have expanded our description of the scoring method for *pals-5p::GFP*; detailed in the section *"Fluorescent reporters for viral infection"* in Material and Methods.

Additionally, for a clearer visual representation, we have included in Supplementary Figure 2 representative images of the *pals-5p::GFP* reporter in mock- or virus-inoculated ERT54 animals grown on the key bacterial environments.

4. Supplementary Figure 2A- it is not clear why the y-axis is different between A and B. Are they both supposed to be percentage of animals showing pals-5 reporter activation? If so, why are the percentages so low in 2A (<2%) compared to 2B (~40-80%)?
There was an error and we corrected the label. The y-axis in 2A represents the relative rate compared with the mean infection levels on OP50 in each block.

5. I suggest re-reviewing the manuscript for grammatical errors and sentence structure throughout. For example, in the abstract line 17- naturally should be "natural" and in the Introduction line 33 "a key role in shaping various of its traits" should be re-worded.
We have thoroughly reviewed the manuscript and made necessary corrections.

6. Several of the figure labels are illegible (e.g. Figure 2A-C, Supplementary Figure 3A).
We have addressed the issue and the figures have been corrected to ensure legibility. Please note that we have used the PACE tool suggested by PLOS to transform the figures into the appropriate format. However, after the site builds the PDF the images look slightly blurred; the images look properly when they are downloaded (upper right, "Click here to access/download;Figure;Figure X.tif")

7. The authors identify a dramatic difference between Leucobacter luti BIG0106 and JUb18 strains relating to their effects on pals-5 reporter activation during OV infection but do not provide a possible explanation for this difference. The authors should ensure the identity of those bacterial strains are verified.
We appreciate the reviewer's concern and thank them for helping correct an error. We have verified the identity of these two bacterial strains, as well as the five other natural strains further characterized, using 16S sequencing. This process confirmed the identification of the five key strains. However, we detected contamination in our *Leucobacter luti* stocks. After ensuring the proper isolation of the correct species, we re-evaluated their influence on viral susceptibility. Our findings indicated that the previously observed differences were due to the contamination. Consequently, we have updated the values, and removed corresponding mentions in the text.

8. For the experiment in Figure 2D where two OV strains are compared, how did the authors ensure they were plating out similar doses of the two viruses? Also in Fig. 2, it is not clear what "Experiment 1 2 3" is referring to at the bottom.
We indeed did not ensure that similar doses of infective virions were inoculated (see difficulties in Frézal et al. 2019).. Hence, we removed the comparison between the two viral strains. The purpose of this test was to observe the effects of bacteria on two different viral preparations of distinct virus genotypes in case of a highly specific interaction. Both the figure and the accompanying text have been updated to reflect this.

9. In Fig. 2E, the authors argue that because one bacterial strain (BIGb0102) that enhanced OV infection rates leads to higher brood sizes that bacteria that enhance infection cannot be doing so by generally weakening host physiology. This cannot be concluded on the basis of this one example, because it is possible that some of the bacterial strains that enhance infection and that reduce worm brood sizes (e.g. BIGb0172) do so by reducing animal fitness. This relates to my earlier point regarding whether these different bacteria affect worm development rates compared to OP50 because altered development may lead to reduced brood sizes. Was brood size determined for each bacterial isolate in the absence of OV as well? Details regarding how brood size was measured should be included.
We agreee that it is possible that the developmental rate change on a specific bacterial strain may affect viral infection (either way), however our results do not provide much evidence in favor of this hypothesis and clearly rule this out as a general mechanism explaining the relative effects of the six bacteria.

We removed the statement about weakening host physiology. The figure provides brood size data on each bacterial isolate without OV. We have revised the manuscript to give a more comprehensive description of this experiment.

10. Line 232 refers to Supplementary Figure 6C but I think this should be 5C. Also line 245 refers to Supplementary Figure 7 but there is figure does not exist so I think this should be Supplementary Figure 6. In general, references to each figure in the text should be double-checked throughout.
We have double-checked and corrected all figure references throughout the manuscript.

11. The bars in Fig. 6G that are supposed to indicate WT or drh-1 genotypes look the same to me, precluding interpretation of these data.
We have rectified the error in display of the figures, and it should now be easy to distinguish the bars representing each genotype.

REVIEWER 3

1. Line 169: Figure 7B should be 3B. Line 242: 7G should be 6F. Line 245: Supplementary Figure 7 should be 6. The authors should double check Figure numbers throughout.
References to each figure have been checked throughout and corrected.

2. The definition of “natural” bacteria should be better defined. It seems odd to call E. coli “non-natural” bacteria (line 257).
We agree. We refer to bacteria found to be associated with the nematode in natural conditions. This has been clarified both in the title of the manuscript: *“**Naturally-associated bacteria** modulate Orsay virus infection of Caenorhabditis elegans”* and in the text: *“In this study, we investigated the effect of bacterial environments on C. elegans susceptibility to OrV. We focused on **monocultures of bacterial clones isolated from C. elegans natural habitats** (14, 15, 16, 36, 37), **which for short will be referred to as "natural bacteria**”*.
We avoided the term non-natural for *E. coli*: *“… the majority of the tested natural bacteria tested reduced host susceptibility to viral infection compared to the **E. coli OP50 environment commonly used in laboratories.**”*

3. Link to the full strain list (Table S2) needs to be mentioned in the first subsection of the Material and Methods.
There was a already a mention to Table S2 in the text: *“C. elegans nematodes were cultured on Nematode Growth Media (NGM) plates at 20°C and C. briggsae animals at 23°C, following*

standard procedures (54, 55). The NGM plates were seeded with 100 µL of a monobacterial culture. ***The nematode strains used in this study are listed in Supplementary Table 2****".*

4. Line 304: "It was created…" suggests a new strain was generated. Details on how to validate genotype (et cetera) are missing.
Indeed, a new strain was generated.
We added the PCR primers used to validate the genotype: "*The primers used to confirm the deletions were AAACTCGCCTGACGGATGAG, TTGGAACTGAGCGATTGGCA, and TCGGTACCTTCGACTAGCAAC.*"

5. Line 340: "Evaluation of viral infection" should mention FISH.
The mention of FISH was added.

6. S4 uses a colorblind unfriendly palette.
Problems in the rendering of the figures may have made it look difficult, but we have tested that the palette (used through all the manuscript) is colorblind friendly. In addition, in the panels of Figure 3A involving lines, we have changed the shape of each data point according to the bacteria so it facilitates the interpretation of the figure for colorblind readers.

7. Fig. 1 use of terms enhancer and suppressor are not strictly known and inaccurate.
We have clarified how we use these terms. A phenotype can be either genetically or environmentally modified.

8. "Worms" is jargon and has an inherent negative connotation that diminishes the impact of C. elegans research; nematodes, C. elegans, or animals are better descriptors.
We agree. This has been changed.
